# Feasibility and Preliminary Efficacy of a New Online Self-Help Intervention for Depression among Korean College Students’ Families

**DOI:** 10.3390/ijerph19042142

**Published:** 2022-02-14

**Authors:** Minji Gil, Suk-Sun Kim

**Affiliations:** 1 Graduate School, College of Nursing, Ewha Womans University, Seoul 03760, Korea; zoemjgil@gmail.com; 2 Ewha Research Institute of Nursing Science, College of Nursing, Ewha Womans University, Seoul 03760, Korea

**Keywords:** depression, e-health, family therapy, mental health promotion, suicide

## Abstract

Applying innovative online approaches to interventions for preventing depression is necessary. Since depressive emotions are typically shared within the family, the development of interventions involving family members is critical. This study thus aimed to examine the feasibility, acceptability, and preliminary outcomes of a new online self-help intervention, MindGuide, among Korean college students’ families. We developed MindGuide, which integrates cognitive behavioral therapy with mindfulness and an emotional regulation approach. A one-group pretest–posttest design was used to measure the changes in the Center for Epidemiological Studies Depression Scale, the Attitude Toward Suicide scale, and the Satisfaction With Life Scale before and after the intervention. Of the 34 families that began the program, completion rates were 88.2%, 85.3%, and 91.2% for fathers, mothers, and children, respectively. The findings indicated that the MindGuide program is feasible and acceptable for families of Korean college students. The results support the potential effect of MindGuide on reducing depression, improving positive attitudes toward suicide prevention, and enhancing family relationships in participants at risk of depression. However, future research is needed to thoroughly explore and evaluate the efficacy of the MindGuide program.

## 1. Introduction

Depression is one of the most common mental illnesses that increases the mental health-related disease burden globally and the risk of suicide [1,2]. In Korea, the prevalence rate of depression was 36.8% in 2020, the highest rate among Organization for Economic Co-operation and Development (OECD) members [3]. Although more than 613,000 Koreans are in need of medical treatment for depression, about 75% of them never seek appropriate treatment due to a lack of awareness about mental disorder, and stigmatization of mental disorder, which contributes to high suicide rates [4].

Moreover, an increasing trend of depression has been observed among people in their 20s and middle adults in Korea [4]. Suicide is the leading cause of death among people aged 10–39 years and the second highest in those aged 40–59 years [5]. However, college students and middle-aged adults in Korea have not received sufficient attention despite their vulnerability to depression and suicide. Preventing or delaying the onset of depression is an important way to reduce its burden [6]. Although preventive interventions for depression are vast and extensive, it is vital that they are tailored for particular target groups (e.g., college students and middle-aged adults) [6].

Considering the busy lives of Korean college students’ and middle-aged parents, it is necessary to apply innovative online approaches as interventions to prevent depression. Online interventions can provide opportunities for easy access to mental healthcare in an earlier stage of depression without the financial, geographic, and time limits that traditional offline interventions have [7,8,9]. In addition, it may be a cost-effective intervention for reducing depression [10,11]. To illustrate, previous research has emphasized that online interventions are effective in reducing depressive symptoms [7,8,9,12,13], as well as the stigma of mental illness, which fosters openness toward seeking mental healthcare [8]. Furthermore, several systematic reviews and meta-analytic studies have demonstrated substantial evidence for the effects of internet-based cognitive behavioral therapy (CBT) for depression administered with or without personal guidance [7,8,12,13,14,15].

Furthermore, previous studies have investigated the meaning of culture for expressing depressive symptoms and found that culture shapes the symptoms and expressions of depression [16]. Koreans tend to suppress emotions and do not express their feelings to others in a public place because of the traditional Korean culture of Confucianism [17,18]. In particular, Koreans with depression express less depressed mood and more hypochondriasis and suicidality than Americans, which may lead to the underdiagnosis of depression and delay adequate treatment [19]. Thus, considering Koreans’ emotional characteristics, online integrative emotional behavior (IEB) interventions, including strategies for dealing with distressing thoughts, emotions, and behaviors, are the best-suited to prevent depression among Korean college students and middle-aged adults [20,21,22].

Since people in collectivist cultures such as China, Japan, and Korea emphasize social relationships, including family, friends, and neighbors, depression is associated with social relationships, including loneliness, social support, and frequency of social contact [23,24,25]. In particular, depressive emotions are typically shared within families [25,26]. Thus, the development of interventions that involve family members is critical. Two systematic reviews found that family-oriented interventions for adult mental health treatment and depression in older adults improved patient symptoms, family functioning, treatment engagement and reduced relapse rates [27,28,29].

However, most family-oriented interventions included patient–caregiver dyads, not family triads (fathers, mothers, and children). Therefore, the purpose of this study was to explore the feasibility of recruiting and retaining family triads, as well as the acceptability of the new online IEB intervention, MindGuide, we developed, with Korean college students’ families. In addition, we evaluated the preliminary outcomes (e.g., depression, attitude toward suicide prevention, and satisfaction in life) of the intervention in two subgroups formed by the Center for Epidemiological Studies Depression Scale (CES-D) cutoff score.

## 2. Materials and Methods

### 2.1. Research Design

This study used a single-group pretest–posttest design and a mixed method. The Ewha Womans University IRB (IRB#: 126-15) approved this study, and informed consent was obtained from the participants prior to data collection.

### 2.2. Participants and Setting

As MindGuide was eventually developed to prevent depression and suicide among Korean adults in the context of family, we recruited Korean college students’ families consisting of fathers, mothers, and children using online advertisements on the MindGuide website and several social media platforms. We have provided the study information and consent form on the MindGuide homepage. If people left their contact information, including names, email addresses, and telephone numbers, the research assistant (RA) contacted them by telephone to screen for eligibility. The inclusion criteria were as follows: (a) having a 19–29-year-old college student; (b) all family members (father, mother, and a college student) agreeing to participate; and (c) being able to use email and the Internet with a mobile device (e.g., smartphone or tablet). We excluded families who (a) had divorced or separated parents; and (b) had a family member who was diagnosed with depression or other mental diseases and was currently receiving drug treatment or other psychotherapy. This is because taking antidepressants or other psychotherapeutic drugs may influence the effectiveness of the intervention. The RA contacted each family member separately and explained the study to them.

If all family members met all eligibility criteria, they received a welcome email with a MindGuide ID, which included a family ID number and individual number. During the first visit to the online MindGuide program, each family member created an account using the provided ID number and completed the registration process, including online informed consent and the baseline survey. They were then able to log on to the MindGuide program at any time until they completed the program modules. We offered $90 incentives to each family member to complete the program.

### 2.3. Intervention: The MindGuide Program

MindGuide (http://mindguide.kr, accessed on 12 February 2022) is a new online self-help intervention that focuses on promoting mental health and preventing depression in college students’ families through the integration of CBT [30], mindfulness [31], and the emotional regulation approach [32]. The project team consisted of researchers, psychiatric mental health nurse practitioners working at the mental health welfare center, the hospital, the university, a web designer, and technical programmers. The MindGuide program was developed by systematically reviewing interventions related to suicide and depression prevention. The MindGuide intervention includes eight modules that educate participants on how to gradually manage depression and their emotions, as well as to pursue personal happiness based on their personal values (Table 1). Each module comprises three sessions that takes no longer than 60 min to complete, while each session takes approximately 15–20 min to complete, depending on individual learning abilities.

The MindGuide program provides psychoeducation and activities through an illustrated narrative with CBT techniques for challenging and restructuring cognitions; mindfulness for helping clients learn how to accept and detach from thoughts and feelings; and emotional regulation techniques for labeling, reappraisal, and expressing emotions. This program seeks to reduce uncomfortable emotions and thoughts while encouraging participants to accept these experiences and emphasizing valued living, to move forward in the direction of their values, and to promote psychological flexibility.

The program was designed to suit Korean students’ family members using family case studies. It uses a case study teaching method to promote an understanding of the connections between topics and everyday life. Furthermore, it suggests homework tasks between sessions to encourage the practice of what was learned in each session. Each session consisted of text, illustrations, mindfulness audios, activities, short videos, and practical exercises.

The content validity of the MindGuide program was tested by five experts (one psychiatric doctor, two psychiatric and mental health nursing professors, one clinical psychologist, and one family counselor) and revised based on their feedback. Experts were requested to rate the content validity in each module on a four-point scale ranging from 1 to 4, with a higher score indicating extremely helpful content. After the experts individually scored each module, the content validity index of all modules ranged from 0.80 to 1.00, with an average of 0.98, indicating high validity. These models were adopted as the interventions.

The program was developed on a mobile responsive website, so that it can be accessed using a computer, tablet, or mobile phone at any time and place. Participants completed 24 sessions and activities sequentially twice a week for three months, and all data were collected online. The participants received an incentive when their participation was terminated.

### 2.4. Data Collection

Data were collected between March 2017 and November 2017. Feasibility, acceptability, and preliminary outcomes of the intervention were assessed both qualitatively and quantitatively. Feasibility refers to participant recruitment, retention, and completion rates [33]. Acceptability refers to how well participants are satisfied with the MindGuide program, particularly the usability of the online intervention [34].

#### 2.4.1. Qualitative Data

To evaluate the feasibility and acceptability of MindGuide, the participants were asked to respond to questionnaires and semi-structured interviews after completing the program. Interviews lasted 30–60 min and were audio-recorded and transcribed for the data to be analyzed. Participants were asked questions such as, “Do you think MindGuide would be effective to prevent depression?” “What content and activities are you satisfied with the program?” and “How acceptable do you feel it is to participate in the program?”

#### 2.4.2. Quantitative Data

We evaluated the rate of program completion and the participants’ perceptions of the program’s impact. Program satisfaction was measured using a five-point scale from 1 to 5; a higher score indicated greater satisfaction. Depression, attitude toward suicide prevention, and life satisfaction were measured at baseline and after the intervention to evaluate the effectiveness of the intervention.

Depression was measured using the 20-item CES-D [35]. Each item was scored on a four-point scale (0–3) and the total score ranged from 0 to 60; a higher score indicated a higher level of depression. A cutoff score of 16 or greater reflects individuals at risk for clinical depression [35]. Two subgroups (normal and risk groups) were formed based on the CES-D cutoff score. Cronbach’s alpha at baseline was 0.95.

Attitude toward suicide prevention was measured using the Attitudes Towards Suicide (ATTS) scale [36]. We found this scale by searching the literature to identify existing measures of attitudes towards suicide prevention. However, this scale includes items relating to suicide prevention myths that do not suit Korean culture [37,38]. Therefore, we only used 14 items (1, 6, 9, 11, 12, 13, 22, 23, 24, 26, 27, 30, 31, 37) to represent attitudes toward suicide prevention [38]. Each item was scored on a five-point scale (1–5), with higher total scores indicating an adaptive attitude toward suicide prevention. An adaptive attitude toward suicide prevention refers to whether individuals believe suicide is preventable, whether it is important to intervene with at-risk individuals, and whether seeking help for mental illness is viewed as a form of self-care [39]. Cronbach’s alpha at baseline was 0.67.

Satisfaction with life was measured using the Satisfaction With Life Scale (SWLS) [40]. The SWLS is a five-item scale that measures cognitive judgment of satisfaction with one’s life. Each item was scored on a seven-point scale (1–7) and total scores ranged from 5 to 35; a higher score indicates that respondents love their lives and feel that things are going well [40]. Cronbach’s alpha for the baseline was 0.91.

### 2.5. Data Analysis

The participants’ characteristics were analyzed using descriptive statistics. Differences between completers and non-completers were assessed using the chi-square and Mann–Whitney tests for small sample sizes. In addition, differences in study variables between family members were evaluated using analysis of variances (ANOVA). All analyses were performed using SPSS version 27 (IBM, Armonk, NY, USA).

Feasibility was examined by the number of people logged into the program homepage and the completion rate of each module and the entire MindGuide program. To explore acceptability and perceived outcomes, the responses to the questionnaires and semi-structured interviews were analyzed using content analysis [41]. The answers that the participants wrote in the MindGuide program were extracted in an Excel file. In addition, data from semi-structured interviews were transcribed. Two researchers read the narratives separately to create codes that reflected the content. Subsequently, they created categories and themes from these codes. When there was a disagreement between the researchers, resolution was achieved through discussion.

Changes in the preliminary outcome variables between baseline and post-intervention were evaluated using the within-group effect size and Cohen’s *d* for a small sample size. Cohen’s *d* was calculated by subtracting the post-mean from the baseline mean and dividing this by the pooled standard deviation of the two means. Effect sizes of ≤0.20, ≥0.50, and ≥0.80 were considered to be small, medium, and large, respectively [42]. Outcomes were analyzed by two groups (normal and risk groups) according to CES-D cutoff score (≥16) [35].

## 3. Results

### 3.1. Sample Characteristics

The final sample for all analyses comprised 90 individuals: 29 mothers (32.2%), 30 fathers (33.3%), and 31 children (34.5%). The baseline participant characteristics are presented in Table 2. Mean age was 52.85 ± 4.23 for fathers, 50.26 ± 4.14 for mothers, and 21.53 ± 2.29 for children. The majority (57.28%) of the participants were women.

The mean income satisfaction was 3.03 ± 0.67 for fathers, 2.94 ± 0.86 for mothers, and 3.32 ± 0.88 for children. There were no significant differences regarding satisfaction income, CES-D scores, and SWLS scores, but there was a statistically significant difference in ATTS scores (*F* = 6.97, *p* = 0.001) among the groups. In addition, the findings of the post-hoc tests indicated that fathers had significantly lower ATTS scores than mothers and children.

### 3.2. Feasibility

**Figure 1:**
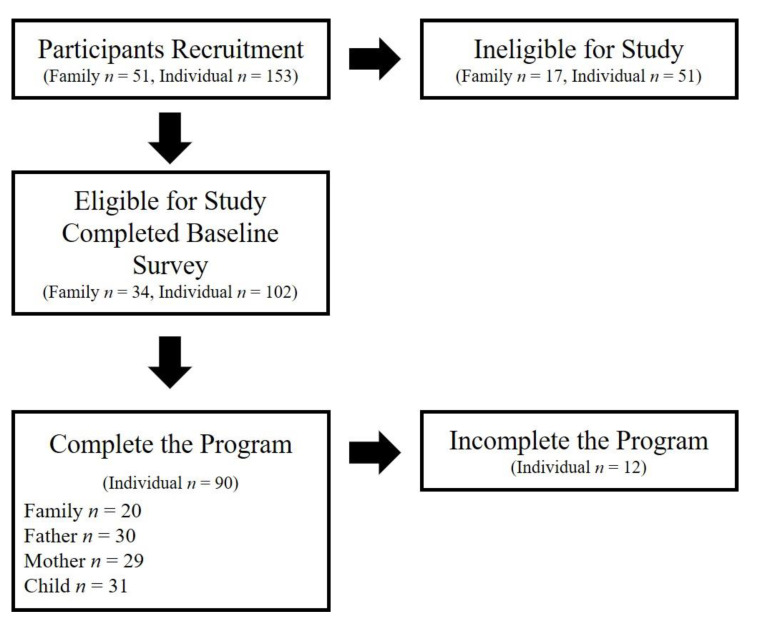
Flow diagram of participants entering into MindGuide.

The flow of participants through the program is shown in Figure 1. Of the 51 families (153 individuals) recruited, 34 (102 individuals, 67.7%) that met the inclusion criteria were included in the study. Seventeen families (51 individuals, 33.3%) were excluded because at least one family member did not agree to participate in the MindGuide program. Of the 102 participants who logged on to the MindGuide website, provided consent, and completed the baseline survey, 90 participants (88.2%) completed all the modules and the post-survey. Twelve participants did not complete the program because they were busy and less motivated.

The completion rates were 88.2%, 85.3%, and 91.2% for fathers, mothers, and children, respectively (Table 2). Twelve participants (11.8%), including four fathers, five mothers, and three children, did not complete the program. Compared to non-completers, completers did not differ regarding gender (χ2 (1) = 0.43, *p* = 0.51) and depression (Mann–Whitney U = 534, *p* = 0.95).

### 3.3. Acceptability

After completing the MindGuide program, the majority (81.1%) of participants reported satisfaction with it (Table 2). In the interviews, most participants were satisfied with the online delivery method of the program. One participant said, “The online program was easy to use and convenient to access through a smartphone.” Another mentioned, “I liked this online program because I could access it at any time and place.”

The majority of participants (82.2%) reported satisfaction with family case-based learning and activities (e.g., writing their own stories, taking a quiz, or uploading photos). One mother stated, “It was helpful to me to have specific examples on how to be aware of my feelings and to write them down. I could apply what I learned through the program to my daily lives.” The other said, “It was great to take a photo to memorize the moment when my husband and I wrote down what happiness is and what we could do for that.”

However, a few (5.6%) did not find some of the teaching resources to be acceptable. One participant mentioned that the materials presented in the text and illustration could be boring and that audio, or video clips, might have been more preferable. A few participants experienced technical errors when they participated in the program. Moreover, some participants suggested that receiving feedback from research teams through email or phone calls during the program might be helpful in keeping their interest and attention to complete the program.

### 3.4. Perceived Outcomes

Most participants (92.2%) thought MindGuide helped them reduce their depressed mood by learning how to be aware of and express their feelings. In addition, they reported increased calmness and relaxation by practicing mindfulness. Some participants stated that the program made them more positive, so they were able to look at life more positively.

Participants (48.9%) believed that the program helped them create a more positive family relationship. They realized the importance of family and practiced expressing love for their families. Participants stated that learning communication techniques allowed them to express their feelings and thoughts consciously to family members, thus helping to enhance family relationships. A mother reported, “I used to be angry at the son without telling him why I was angry. However, through the program, I learned a lot about my communication skills. Now I am able to talk about my depressive mood to my family.”

### 3.5. Preliminary Outcomes

The means and standard deviations of the study variables and within-group effect sizes (Cohen’s *d*) are shown in Table 3. For the baseline–post-program comparison on CES-D, the effect size indicated that participants in the risk group experienced medium to large reductions in depression: father (*d* = 0.32), mother (*d* = 0.69), and child (*d* = 0.52). However, the participants in the normal group perceived no change (*d* = 0.16~0.10).

All participant reports on the ATTS indicated that they experienced improved adaptive attitudes toward suicide prevention with medium to large within-group effect sizes (*d* = 0.25~0.80). While the father in the risk group (*d* = 0.29) and the mother in the normal group (*d* = 0.25) reported a slight improvement in life satisfaction, the father in the normal group, the mother in the risk group, and the child experienced no change.

## 4. Discussion

This study evaluated the feasibility, acceptability, and potential effects of online self-help IEB interventions, the MindGuide program, with 34 Korean college students and their middle-aged parents. The findings indicated that the MindGuide program was feasible and acceptable for families of Korean college students. In addition, the results support the potential effects of MindGuide in reducing depression, improving positive attitudes toward suicide prevention, and enhancing family relationships.

Our study demonstrated that we were able to recruit, enroll, and retain 34 family triads, including middle-aged fathers, mothers, and college students, for the MindGuide program. Although a fair number of families (33.3%) were excluded during screening for eligibility, the completion rate of the entire program (88%) was high. Especially, children had higher completion rates than parents. This is not congruent with the results of a systematic review study [43] which revealed that online mental health interventions tend to have relatively low completion rates. In particular, unsupported online interventions tend to have relatively poor adherence and high attrition rates [44]. However, although MindGuide is an online self-help program without support, it had a high completion rate. We assume that participating in the program as a family triad would improve overall completion rates because family members support each other to finish the program.

Our findings suggest that the MindGuide program is acceptable to Korean college students’ families. All participants generally expressed satisfaction with the MindGuide program. In particular, they were satisfied with the learning family case study, which helped them to understand another family member and to apply what they learned in their daily lives. Moreover, training family members via the internet offered many potential benefits. Participants reported that they could easily access the program at any time and place through the mobile responsive website: http://mindguide.kr/.(accessed on 12 February 2022) They also participated in the program in their own time and at their own pace. This is similar to findings from a systematic review and meta-analysis, which reported that online mental health interventions are easily accessible and widely disseminated at a low cost [8].

However, some elements of MindGuide, such as technical errors and lack of coaching support, were not highly acceptable. These results reflect previous findings that lack of motivation and technical problems were reasons for drop out [45]. To make MindGuide more accessible, future research is needed to compare the effectiveness of the MindGuide program with or without coaching support and technical alarms.

After participating in the MindGuide program, the participants thought that the program helped them to reduce their depressed mood and create more positive family relationships by realizing the value of family and learning communication techniques. Most family-based preventive interventions primarily target the prevention of mental problems (e.g., depression, substance use) by strengthening family relationships and resilience [46,47]. Similarly, the participants experienced reduced depression and enhanced family relationships through the MindGuide program.

The current study highlights the potential benefits of the MindGuide program on depression, especially in the risk group (CES-D ≥ 16). Participants’ depressive symptoms in the risk group were reduced after completing the MindGuide program, and the effect size was medium to large. Conversely, we found no significant change in depression in the control group (CESD < 16). The results suggest that participants at risk for clinical depression might benefit more from the MindGuide program for reducing depression than those who are not at risk.

Moreover, all participants experienced improved positive attitudes toward suicide prevention, with medium to large effect sizes. This finding is consistent with previous research showing that family suicide care educational intervention is effective in increasing suicidal caring ability and positive attitudes toward their suicidal family members [48]. One possible explanation for this result is that the MindGuide program may help participants understand how people with suicidal ideation feel and, in turn, create more positive attitudes towards them. Another interpretation is that the MindGuide program may change positive attitudes toward suicide prevention by strengthening the sense of family belonging and connectedness to the family [49].

Due to several limitations related to the single-group pretest–posttest design and the small sample size, the findings of this study should be interpreted with caution. Future research with a control group and a larger sample is needed to obtain further insight into the effectiveness of the MindGuide program on mental health. In addition, despite collecting family triad data, we could not analyze the data at the family level because of the small sample size. Additional studies with a large sample size should thus consider data analysis at the family level. Finally, convenience sampling, which was used to recruit Korean college students’ family triads, also limits the generalizability of the results to older adults, some of whom may not be well educated. This suggests a need for future randomized controlled trials with diverse populations. Despite these limitations, one of the strengths of this study is that we used a mixed method methodology to obtain more in-depth information about whether MindGuide is feasible, acceptable, and effective.

## 5. Conclusions

The study demonstrates that MindGuide, a new online self-help IEB intervention, is feasible, acceptable, and effective in reducing depression and improving positive attitudes toward suicide prevention for Korean college students’ families. Although the effect size of depression was medium to large only in the risk group, most participants experienced that the MindGuide program helped to decrease depression and strengthen family relationships. This suggests that MindGuide could be used as a vehicle to prevent depression and suicide.

## Figures and Tables

**Table 1:** The MindGuide program.

Modules	Module Titles	Session Titles
1	Knowing and preventing depression	To know depression To recognize depressed feelings To prevent depression
2	Mindfulness	To know the mindfulness To know one’s feeling To love oneself
3	Managing emotions	To recognize emotions To uncover hidden emotions To express emotions
4	Satisfying conversation	To know types of communication To speak with respect To empathic listening
5	Recognizing thoughts	To look back on one’s thoughts To identify unhelpful thoughts To find thinking-feeling connection
6	Finding happiness	To find values To find balance and happiness To pursue happiness
7	An adventure to find “pleasure”	To where did pleasure go? To the pleasure’s magical staff To the tower of labyrinth
8	Protecting and respect for all life	To recognize warning signs of suicide To assess suicide risks To work together

**Table 2:** Baseline characteristics and completion rate of the program (*N* = 102).

Variables	All *n* (%)/M ± SD	Father *n* (%)/M ± SD	Mother *n* (%)/M ± SD	Child *n* (%)/M ± SD	*F (p)*
Baseline characteristics	102 (100%)	34 (100%)	34 (100%)	34 (100%)	
Gender					
Male	43 (42.2%)	34 (100%)		9 (26.5%)	
Female	59 (57.8%)		34 (100%)	25 (73.5%)	
Age (year)	41.55 ± 4.72	52.85 ± 4.23	50.26 ± 4.14	21.53 ± 2.29	
Education					
High school	47 (46.1%)	6 (17.7%)	7 (20.5%)	34 (100%)	
Associate degree	6 (5.9%)		6 (17.7%)		
Bachelor’s degree	34 (33.3%)	18 (52.9%)	16 (47.1%)		
Graduate degree	15 (14.7%)	10 (29.4%)	5 (14.7%)		
Religion					
Protestant	56 (54.9%)	19 (55.9%)	19 (55.9%)	18 (52.9%)	
Catholic	18 (17.6%)	5 (14.7%)	8 (23.5%)	5 (14.7%)	
Buddhist	5 (4.9%)	2 (5.9%)	3 (8.8%)	1 (2.9%)	
None	22 (21.6%)	8 (23.5%)	4 (11.8%)	10 (29.4%)	
Income satisfaction	3.10 ± 0.81	3.03 ± 0.67	2.94 ± 0.86	3.32 ± 0.88	2.10 (*p* = 0.128)
Study variables					
CES-D	9.96 ± 10.08	8.38 ± 9.54	8.35 ± 9.73	13.15 ± 10.46	2.63 (*p* = 0.077)
ATTS	50.47 ± 5.91	47.48 ± 4.94	51.88 ± 6.65	51.94 ± 4.98	6.97 (*p* = 0.001)
SWLS	21.88 ± 5.55	21.15 ± 5.68	21.68 ± 5.56	22.82 ± 5.42	0.809 (*p* = 0.448)
Completion rate					
Module 1	99 (97%)	32 (94.1%)	33 (97%)	34 (100%)	
Module 2	97 (95.1%)	32 (94.1%)	32 (94.1%)	33 (97%)	
Module 3	95 (93.1%)	31 (91.2%)	32 (94.1%)	32 (94.1%)	
Module 4	93 (91.2%)	30 (88.2%)	31 (91.2%)	32 (94.1%)	
Module 5	91 (89.2%)	30 (88.2%)	30 (88.2%)	31 (91.2%)	
Module 6~8	90 (88.2%)	30 (88.2%)	29 (85.3%)	31 (91.2%)	
Program Satisfaction	90 (100%)	30 (100%)	29 (100%)	31 (100%)	
Satisfied	79 (81.1%)	23 (76.7%)	24 (82.8%)	26 (83.9%)	
Neutral	12 (13.3%)	6 (20.0%)	3 (10.3%)	3 (9.7%)	
Unsatisfied	5 (5.6%)	1 (3.3%)	2 (6.9%)	2 (6.4%))	

Note: CES-D: Center for Epidemiological Studies Depression Scale; ATTS: Attitude Toward Suicide Scale; SWLS: Satisfaction With Life Scale.

**Table 3:** Preliminary outcomes (*N* = 90).

Variables	Group	Baseline	Post-Program	Effect Sizes Baseline to Post-Program
		M ± SD		
CES-D	Father			
	Normal group (*n* = 22)	3.48 ± 3.17	4.13 ± 4.68	0.16
	Risk group (*n* = 7)	25.43 ± 4.50	23.86 ± 5.36	0.32
	Mother			
	Normal group (*n* = 25)	5.60 ± 4.30	6.08 ± 5.23	0.10
	Risk group (*n* = 4)	28.50 ± 13.53	20.75 ± 8.34	0.69
	Child			
	Normal group (*n* = 22)	7.32 ± 4.64	8.32 ± 10.82	0.12
	Risk group (*n* = 9)	25.00 ± 9.68	19.22 ± 12.57	0.52
ATTS	Father			
	Normal group (*n* = 22)	47.83 ± 5.07	52.13 ± 5.62	0.80
	Risk group (*n* = 7)	46.29 ± 5.65	47.68 ± 4.65	0.25
	Mother			
	Normal group (*n* = 25)	51.16 ± 6.82	55.28 ± 5.98	0.64
	Risk group (*n* = 4)	51.50 ± 5.26	52.75 ± 2.50	0.30
	Child			
	Normal group (*n* = 22)	53.00 ± 4.97	56.27 ± 6.67	0.56
	Risk group (*n* = 9)	49.44 ± 4.13	52.78 ± 6.14	0.64
SWLS	Father			
	Normal group (*n* = 22)	22.82 ± 5.01	23.70 ± 4.04	0.19
	Risk group (*n* = 7)	15.43 ± 2.22	16.43 ± 4.35	0.29
	Mother			
	Normal group (*n* = 25)	22.32 ± 5.91	23.68 ± 5.15	0.25
	Risk group (*n* = 4)	17.25 ± 3.40	16.00 ± 0.82	0.51
	Child			
	Normal group (*n* = 22)	24.36 ± 4.22	24.23 ± 4.62	0.03
	Risk group (*n* = 9)	19.67 ± 7.10	20.89 ± 7.66	0.17

Note. CES-D, Center for Epidemiological Studies Depression Scale; ATTS, Attitude Toward Suicide Scale; SWLS, Satisfaction With Life Scale.

## Data Availability

The data presented in this study are available on request from the corresponding author.
